# Deficiency of exocyst complex component Exoc5 exacerbates the progression of kidney fibrosis

**DOI:** 10.1038/s12276-026-01649-8

**Published:** 2026-03-04

**Authors:** Hui Jae Lim, Yong Kwon Han, Mi Ra Noh, You Ri Park, Se Young Jang, Joshua H. Lipschutz, Kwon Moo Park

**Affiliations:** 1 Department of Biomedical Science and BK21 Plus, The Graduate School of Medicine, Kyungpook National University, Daegu, Republic of Korea; 2 Department of Anatomy, School of Medicine, Kyungpook National University, Daegu, Republic of Korea; 3 Cardiovascular Research Institute, Kyungpook National University, Daegu, Republic of Korea; 4 Department of Internal Medicine, School of Medicine, Kyungpook National University, Kyungpook National University Hospital, Daegu, Republic of Korea; 5 Bio-Medical Research Institute, Kyungpook National University Hospital, Daegu, Republic of Korea; 6 Departments of Medicine, Medical University of South Carolina and Ralph H. Johnson Veterans Affairs Medical Center, Charleston, SC USA

**Keywords:** Chronic kidney disease, Experimental models of disease

## Abstract

Fibrosis, an undesirable side effect of the repair process, is due to aberrant cell differentiation and is a typical cause of progressive diseases including chronic kidney disease (CKD). Exocyst complex component 5 (Exoc5), a central component of the highly-conserved eight-protein exocyst complex, is involved in cell differentiation and maturation; however, the role of Exoc5 in fibrosis remains to be defined. Here we investigate the role and underlying molecular mechanisms of Exoc5 on kidney development and fibrosis using kidney proximal tubule cell-specific *Exoc5-*knockout (PT–Exoc5^KO^) mice generated by crossing *Exoc5*^*f/f*^ with *PEPCK-cre* mice. Exoc5-knockout mice showed normal kidney structure and function. Unilateral ureteral obstruction (UUO) led to kidney fibrosis with decreased Exoc5 expression, and Exoc5 knockout worsened the fibrosis. Exoc5 knockout alone increased Yes-associated protein (YAP) expression and exacerbated UUO-induced YAP activation and the expression of CTGF and CYR61, products of YAP, compared with wild-type (PT–Exoc5^WT^) mice. UUO induced paired box 2 (Pax2, which is mainly expressed during kidney development) expression in both mice, and the UUO-induced Pax2 expression in PT–Exoc5^KO^ was greater than in PT–Exoc5^WT^ mice. In HK-2 cells, a human proximal tubule cell, EXOC5 downregulation by siRNA increased YAP and Pax2 expression. EXOC5 downregulation augmented TGF-β-induced YAP activation and epithelial-to-mesenchymal transition. Taken together, our data demonstrate that Exoc5 plays a protective role in kidney fibrosis, implying that Exoc5 could potentially serve as a therapeutic target for the regulation of fibrosis.

## Exoc5 protects against kidney fibrosis through YAP regulation

Fibrosis is a harmful process that can damage organs, including the kidneys. It often leads to chronic kidney disease. Researchers are trying to understand how fibrosis develops and find ways to treat it. A protein complex called the exocyst, which helps transport materials within cells, might play a role in fibrosis. In this study, researchers explored the role of a specific component of the exocyst called Exoc5 in kidney fibrosis. They used mice with a genetic modification that removed Exoc5 from kidney proximal tubule cells. These mice did not show any initial kidney problems but developed worse fibrosis when their kidneys were injured. The researchers found that without Exoc5, there was an increase in processes that lead to fibrosis, such as epithelial-to-mesenchymal transition, where cells change type and contribute to scarring.

This summary was initially drafted using artificial intelligence, then revised and fact-checked by the author.

## Introduction

Fibrosis, an undesirable side effect of the cellular repair process, is critical for the pathogenesis and progression of various diseases^1,2^. The process of fibrosis is associated with a variety of factors, including the accumulation of inflammatory cells, activation of fibroblasts and differentiation of epithelial cells to mesenchymal cells (EMT, epithelial-to-mesenchymal transition)^3,4^. The progression of fibrosis leads to functional and histological defects in organs. In the kidney, fibrosis is a common feature of chronic kidney disease (CKD), which is characterized by a progressive loss of kidney function, ultimately resulting in the need for renal replacement therapy^5,6^. The molecular mechanisms of fibrosis remain to be defined, and there is a critical need for the development of therapeutic interventions.

The exocyst is a highly-conserved octameric protein trafficking complex (Exoc1-8, formerly called Sec3, Sec5, Sec6, Sec8, Sec10, Sec15, Exo70 and Exo84) that was first identified and characterized in budding yeast^7,8^. In earlier studies, it has been shown that the exocyst complex regulates the spatial targeting and docking of vesicles generated in the Golgi complex to specific locations on the plasma membrane^9,10^. The exocyst complex regulates a variety of cellular functions, including: exocytosis, polarity formation, tubulogenesis, epithelia formation and growth, through the control of cellular signaling networks^11–14^. In addition, recent studies have demonstrated that the exocyst complex is implicated in a variety of cellular and organ functions as well as diseases^11,14^.

Exoc5 is centrally located within the exocyst complex and directly connects Exoc6, which is bound to vesicles exiting the trans-Golgi network, to the rest of the exocyst complex already at the specified cellular location. When Exoc5 is dysregulated, the exocyst complex does not function properly in cells, resulting in defects in exocytosis, ciliogenesis, cystogenesis, tubulogenesis and epithelial cell differentiation^12,15,16^. Moreover, data from our lab and others have shown that Exoc5 knockout in distal nephron epithelial cells and the urothelium of the renal pelvis and upper ureter (Exoc5^f/f^;Ksp-Cre mice) causes an obstructive ureter phenotype and premature death in mice^16^; Exoc5 knockout in podocytes (Exoc5^f/f^;Podocin-Cre mice) leads to defects in podocyte foot processes and the slit diaphragm and induces proteinuria and premature death in mice^17^; and in the aortic valve, Exoc5 knockout leads to bicuspid aortic valve and aortic stenosis^15^. In addition, we found that Exoc5 overexpression protects renal tubule cells from injury through EGFR/MAPK activation and the enhancement of epithelial barrier integrity and accelerates the recovery of cultured renal tubular cells from oxidative stress, whereas Exoc5 deletion increases susceptibility to renal ischemia–reperfusion injury^18–20^. Moreover, we showed that Exoc5 is involved in tubulogenesis and epithelial cell differentiation, which are essential for the process of fibrosis^12,19,21,22^.

As our understanding of the exocyst has increased, it has become evident that Exoc5 plays an important role in the progression of fibrosis; however, the mechanism remains to be defined. Therefore, we investigated the role and molecular mechanism of Exoc5 in the development and progression of renal fibrosis. Here, we report that Exoc5 deletion exacerbates the progression of renal fibrosis by the upregulation of EMT and Yes-associated protein (YAP) signaling.

## Materials and methods

### Generation of PT–Exoc5^KO^ mice

Exoc5-floxed (*Exoc5*^*f/f*^) mice were generated as previously described^16^. PEPCK, renal proximal tubule cell-specific Cre recombinase mice, were obtained from University of Nebraska Medical Center^23^. The proximal tubule cell-specific Exoc5-knockout (*Exoc5*^*f/f*^*;PEPCK-Cre*, PT–Exoc5^KO^) mice were generated by crossing *Exoc5*^*f/f*^ mice with *PEPCK-Cre* mice, as described in Supplementary Fig. 1a. *Exoc5*^*f/f*^ littermates were used as controls. The genotyping results confirming floxed and Cre are depicted in Supplementary Fig. 1b.

### Animal experiments

Female mice (10–12 weeks old) weighing 19–22 g were used. All the mice were kept in a specific pathogen-free mouse facility with access to sterilized food, water and bedding under a 12-h–12-h light–dark cycle at 22 °C. To induce unilateral ureteral obstruction (UUO), the mice were anesthetized with pentobarbital sodium (50 mg/kg) intraperitoneally. The right kidney was exposed via a flank incision. The right ureter was completely tied with a 6–0 silk thread, and then the incision was sutured. The body temperature was maintained at 36.4 °C–37 °C during procedures using a surgical heating pad. As, in general, female sex is less susceptible to injury than male, to minimize the association of damage which occurred by UUO, we used female mice^24^. Several mice were administered intraperitoneal injections of 5′-bromo-2′-deoxyuridine (BrdU; 50 mg/kg; Sigma-Aldrich) intraperitoneally. Finally, the mice were euthanized with an overdose of pentobarbital sodium 7 days after UUO, and the kidneys were collected by either snap-freezing in liquid nitrogen for biochemical analysis or perfusion–fixation in PLP (4% paraformaldehyde, 75 mM L-lysine, 10 mM sodium periodate) for histological studies.

### Blood and urine biochemistry

To analyze renal function, blood was collected from mice using a heparinized syringe, and urine samples were collected using metabolic cages. Plasma creatinine (PCr), blood urea nitrogen (BUN), urine creatinine, urine glucose, urine Na^+^ and urine K^+^ were determined using a Vitro 250 Chemistry Analyzer (Johnson and Johnson). Urine osmolality was measured using a cryoscopic osmometer (Osmomat 030-D; Gonotec). Creatinine clearance (CrCl) was calculated.

### Western blot analysis

Western blot was performed as previously described^25^. Antibodies against the following proteins were used: Exoc5 (cat. no. sc-514802), Exoc4 (cat. no. VAM-SV016, StressMarq Biosciences), Exoc6 (cat. no. 12723-1-AP, Proteintech), Exoc7 (cat. no. 28666, Cell Signaling Technology), p-Smad3 (cat. no. ab52903, Abcam), Smad3 (cat. no. 9523, Cell Signaling Technology), β-catenin (cat. no. 8480, Cell Signaling Technology), alpha-smooth muscle actin (α-SMA; cat. no. A2547, Sigma-Aldrich), vimentin (cat. no. 5741, Cell Signaling Technology), Pax2 (cat. no. PRB-276P, Covance), proliferating cell nuclear antigen expression (PCNA; cat. no. m879, DAKO), YAP/TAZ (cat. no. 8418, Cell Signaling Technology), YAP (cat. no. 14074, Cell Signaling Technology), phosphorylated YAP (p-YAP; cat. no. 13008, Cell Signaling Technology), CTGF (cat. no. sc-365970), CYR61 (cat. no. 39382, Cell Signaling Technology), N-cadherin (cat. no. 13116, Cell Signaling Technology) and glyceraldehyde 3-phosphate dehydrogenase (GAPDH; cat. no. NBP600-502, NOVUS).

### Immunofluorescence staining

Following deparaffinization, the sections were incubated in PBS containing 0.2% Triton X-100 (Sigma-Aldrich) for 1 min and then washed in PBS for 10 min. To expose the antigen epitope, sections were boiled in 10 mM sodium citrate buffer (pH 6.0) for 10 min, cooled for 20 min and then washed three times with PBS for 5 min per wash. The sections were blocked with 3% bovine serum albumin in PBS (blocking buffer) for 30 min and then incubated with antibodies against Exoc5 (cat. no. 17593-1-AP, Proteintech), AQP1 (cat. no. MCA2099, Bio-Rad Laboratories), AQP1 (cat. no. ab168387, Abcam), collagen 1 (cat. no. ab21286, Abcam), α-SMA (cat. no. A2547, Sigma-Aldrich), vimentin (cat. no. 5741, Cell Signaling Technology), E-cadherin (cat. no. 3195, Cell Signaling Technology), ZO-1 (cat. no. 21773-1-AP, Proteintech), Pax2 (cat. no. PRB-276P, Covance), PCNA (cat. no. m879, DAKO), BrdU (cat. no. ab6326, Abcam) and YAP (cat. no. 14074, Cell Signaling Technology), at 4 °C overnight. After washing, the sections were incubated with fluorescein isothiocyanate (FITC) or Texas Red-conjugated goat anti-rabbit or anti-mouse IgG and FITC or Cy3-conjugated goat anti-rabbit or anti-mouse IgG for 60 min and then washed three times with PBS for 5 min. Cell nuclei were stained using 4′-6-diamidino-2-phenylindole (DAPI; Sigma-Aldrich). The sections were observed under a Leica microscope (cat. no. DM2500).

### Quantitative real-time PCR analysis

RNA was extracted using NucleoZOL reagent (Macherey-Nagel GmbH KG) from the kidneys. Next, RNA was used for complementary DNA (cDNA) synthesis using the AMPIGENE cDNA Synthesis Kit (Enzo Life Sciences). Quantitative real-time PCR (rtPCR) was performed using the quantitative PCR Green Mix (Enzo Life Sciences) and the Agilent Real-time PCR System (Agilent Technology). The mouse Yap primers were as follows: forward 5′-CCCTCGTTTTGCCATGAACC-3′ and reverse 5′-TCCGTATTGCCTGCCGAAAT-3′. The mouse GAPDH primers were as follows: forward 5′-CATCACTGCCACCCAGAAGACTG-3′ and reverse 5′-ATGCCAGTGAGCTTCCCGTTCAG-3′.

### Immunohistochemical staining

Immunohistochemical staining was performed using antibodies against AQP1 (cat. no. ab168387, Abcam), Na^+^/K^+^ ATPase (cat. no. ab76020, Abcam), SGLT2 (cat. no. 24654-1-AP, Proteintech) and F4/80 (cat. no. MCA497GA, Bio-Rad Laboratories) antibodies. The quantification of F4/80^+^ cells was performed by counting the number of F4/80^+^ cells in multiple randomly selected fields.

### PAS staining

Kidney sections were stained with periodic acid–Schiff (PAS) as described previously^26^. The kidney damage was scored in a blind fashion by investigators as described previously^26^.

### Picrosirius red staining

Kidney sections were fixed with Bouin’s solution at 55°C for 1 h, washed in running tap water until the yellow color disappeared, incubated in 0.1% fast green (Fisher Scientific) for 10 min and washed with 0.5% acetic acid. The sections were then stained with Picrosirius red for 1 h.

### Cell culture

The human renal proximal tubule cell lines (HK-2, ATCC) were cultured in Dulbecco’s modified Eagle medium/nutrient mixture F12 (DMEM/F12) (Thermo Fisher Scientific) containing 5% fetal bovine serum (Thermo Fisher Scientific) and 100 Uml streptomycin–penicillin (WelGENE) at 37 °C in a humidified atmosphere containing 5% CO_2_. The 70–80% confluent HK-2 cells were incubated in Opti-MEM (Thermo Fisher Scientific) without streptomycin–penicillin for 2 h and transfected with 25 or 50 nmol/l of small interfering RNA (siRNA) targeting Exoc5 (Bioneer) and scrambled siRNA (SN-1002, Bioneer) using Lipofectamine 3000 (Thermo Fischer Scientific) for 6 h according to the manufacturer’s instructions. The cell culture medium was changed to DMEM/F12 and further incubated with 5 ng/ml TGF-β (cat. no. 7666-MB-005, R&D Systems) or vehicle for 1 and 48 h.

### Statistics

All data were analyzed using Graphpad Prism 9 software (GraphPad). The results are expressed as the mean ± standard error of the mean (s.e.m.). Statistical analysis was performed using Student’s *t*-test and one-way analysis of variance with Tukey’s post hoc procedure. Statistical significance was set at *P* < 0.05.

## Results

### Exoc5 knockout does not lead to defects in renal development or function

**Fig. 1:**
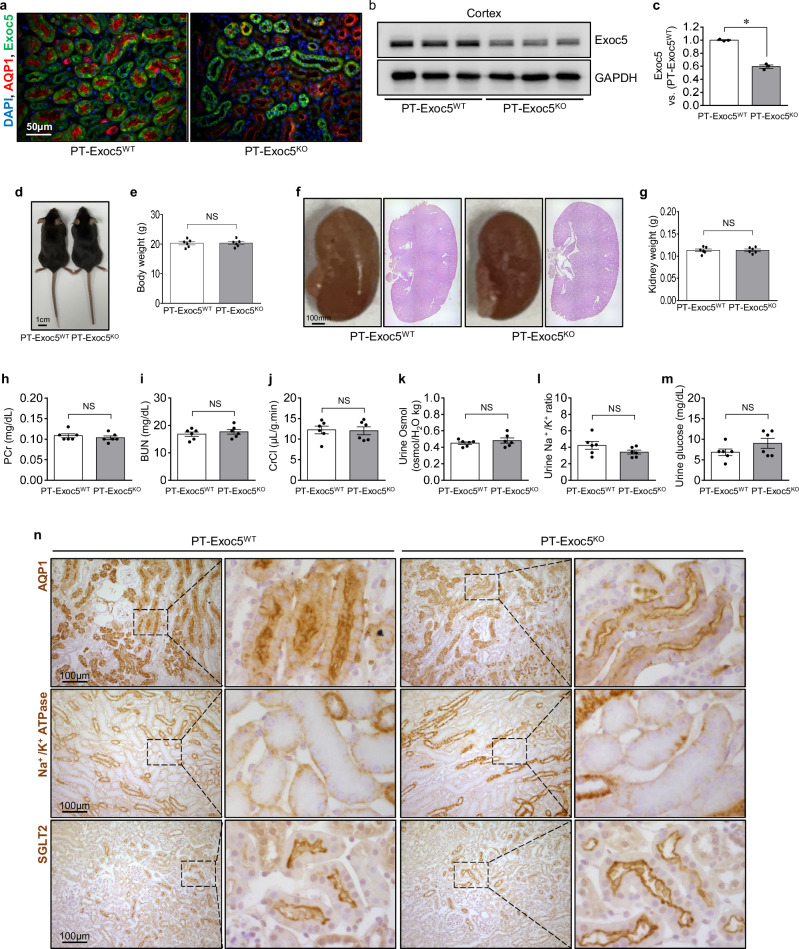
Kidney function in PT–Exoc5^WT^ and PT–Exoc5^KO^ mice. **a** The kidney sections were subjected to immunofluorescence staining using antibodies against Exoc5 (green) and AQP1 (a marker of proximal tubule cells, red). DAPI staining was used to visualize cell nuclei. **b**, **c** The Exoc5 expression in the kidneys of PT–Exoc5^WT^ and PT–Exoc5^KO^ mice was analyzed by western blot. GAPDH was used as the loading control. Densities of bands were quantified using ImageJ software (*n* = 3). **d** Representative images of PT–Exoc5^WT^ and PT–Exoc5^KO^ mice. **e** Body weights of PT–Exoc5^WT^ and PT–Exoc5^KO^ mice. **f** Representative images of kidneys of PT–Exoc5^WT^ and PT–Exoc5^KO^ mice. Kidney sections were subjected to PAS staining. **g** Kidney weights. **h**–**m** PCr (**h**) and BUN (**i**) concentrations, CrCl (**j**), urine osmolality (Osmol) (**k**), urine Na^+^/K^+^ ratio (**l**) and urine glucose concentrations (**m**) were measured. **n** The kidney sections were subjected to immunohistochemical staining using antibodies against AQP1, Na^+^/K^+^ ATPase and SGLT2. Results are expressed as the mean ± s.e.m. (*n* = 4–6). Blood and urine were collected from PT–Exoc5^WT^ and PT–Exoc5^KO^ mice as described in Materials and methods. *P < 0.05. NS, not significant.

To confirm the Exoc5 deletion in the proximal tubule cells, we co-stained the kidney sections using anti-Exoc5 and -aquaporin-1 (AQP-1, a proximal tubule marker) antibodies. In wild-type (PT–Exoc5^WT^) mice, Exoc5 was expressed in all tubule cells, including proximal tubule cells, distal tubule cells and collecting duct cells in the kidney (Fig. 1a). However, in PT–Exoc5^KO^ mice, as expected, Exoc5 was not expressed in AQP1-positive proximal tubule cells (Fig. 1a). In addition, Exoc5 expression in the lysate from the kidney cortex was significantly less in PT–Exoc5^KO^ mice compared with PT–Exoc5^WT^ mice (Fig. 1b, c). These data indicate that proximal tubule-specific Exoc5 knockout mice were successfully generated.

Next, we evaluated whether Exoc5 knockout in proximal tubule cells affects kidney structure and/or function. Exoc5 knockout did not result in defects in body growth (Fig. 1d, e). In addition, Exoc5 knockout did not induce significant anatomical, histological or functional changes in the kidney (Fig. 1f–n); kidney morphology and weights of PT–Exoc5^KO^ mice were no different compared with those of PT–Exoc5^WT^ mice (Fig. 1f, g); PCr, BUN, CrCl, urine osmolality, urine Na^+^/K^+^ ratio and urine glucose levels of PT–Exoc5^KO^ mice were also similar to those of PT–Exoc5^WT^ mice (Fig. 1h–m). The localization of proximal tubule cell transporters was similar in both mice: AQP1 and the sodium-glucose cotransporter 2 (SGLT2) were expressed on the apical surface of the proximal tubule cells, and Na^+^/K^+^ ATPase was expressed at the basolateral membrane of the proximal tubule cells in both PT–Exoc5^KO^ and PT–Exoc5^WT^ mice (Fig. 1n). These results indicate that Exoc5 deletion in the proximal tubule cells does not cause significant defects in polarity, renal function and kidney development.

### Exoc5 knockout augments renal fibrosis following UUO

**Fig. 2:**
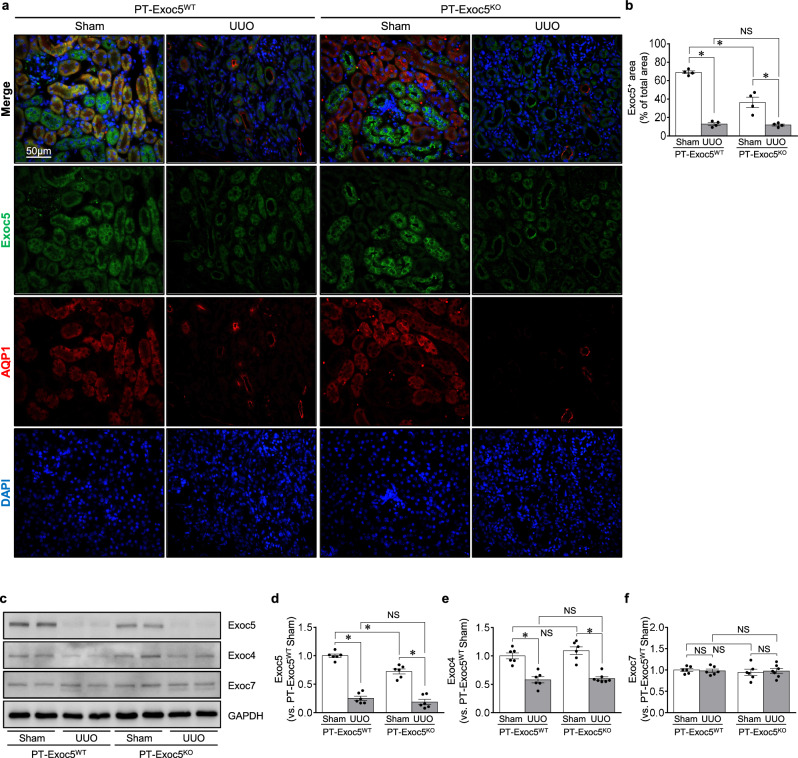

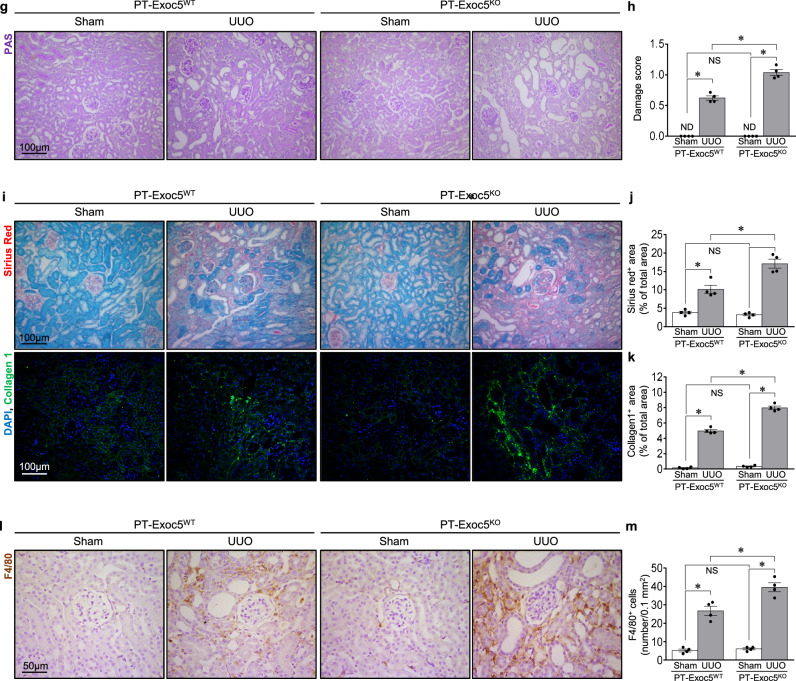
Kidney damage, fibrosis and inflammation in PT–Exoc5^WT^ and PT–Exoc5^KO^ mice following UUO. **a** The kidney sections were subjected to immunofluorescence staining using antibodies against Exoc5 (green) and AQP1 (red). DAPI staining (blue) was performed to visualize cell nuclei. **c**–**f** Western blot (**c**) of the expression of Exoc5 (**d**), Exoc4 (**e**) and Exoc7 (**f**) in normal and UUO kidneys. GAPDH was used as the loading control. Densities of bands were quantified using ImageJ software (*n* = 6). **g**, **i**, **l** The kidney sections were subjected to PAS (**g**) staining, Picrosirius red staining (**i**) and immunostaining using antibodies against collagen 1 (green) and F4/80 (brown) (**l**). DAPI staining (blue) was used to visualize cell nuclei. **h**, The kidney damage was scored as previously described^26^. **b**, **j**, **k** The quantification was performed by measuring the area of Exoc5-, Sirius red- and collagen-1-positivity (*n* = 4). **m** The quantification was performed by counting the number of F4/80-positive cells in a 40× micrograph with a field area of 0.1 mm^2^. PT–Exoc5^WT^ and PT–Exoc5^KO^ mice were subjected to either UUO or sham surgery, and kidneys were collected 7 days following surgery. Results are expressed as the mean ± s.e.m. (*n* = 4). *P < 0.05. NS, not significant; ND, not detected.

We next investigated the effect of Exoc5 deletion on UUO-induced renal fibrosis. UUO decreased the Exoc5 expression in the cells of the proximal tubules, distal tubules and collecting ducts of the kidneys compared with sham operation in both PT–Exoc5^KO^ and PT–Exoc5^WT^ mice (Fig. 2a, b). Similar to the immunostaining data, UUO significantly decreased the amount of Exoc5 expression in the kidney in both PT–Exoc5^KO^ and PT–Exoc5^WT^ mice (Fig. 2c, d). To further define whether Exoc5 deletion affects other the exocysts’ complex expression, we examined the expression of Exoc4, an exocyst subcomplex 1 component, and Exoc7, an exocyst subcomplex 2 component, by western blot analysis. Exoc4 and Exoc7 expression levels in sham-operated PT–Exoc5^KO^ mice were not significantly different compared with those in sham-operated PT–Exoc5^WT^ mice (Fig. 2c, e, f). UUO decreased the expression of Exoc4, but not Exoc7, in both groups compared with their respective sham mice, with no difference between PT–Exoc5^KO^ and PT–Exoc5^WT^ mice (Fig. 2c, e, f). These results show that Exoc5 knockout does not alter the other exocyst components, at least not Exoc4, Exoc6 and Exoc7 expression.

Next, we investigated whether Exoc5 deletion affects renal fibrosis following UUO. UUO resulted in renal tubule cell atrophy, expansion of the interstitium and increases in Picrosirius red and collagen 1-positive areas in both groups of mice, though these changes were greater in PT–Exoc5^KO^ mice compared with PT–Exoc5^WT^ mice (Fig. 2g–k). In addition, UUO increased the number of F4/80^+^ macrophage, which is a major contributor to TGF-β production, in both groups, with a greater increase in PT–Exoc5^KO^ compared with PT–Exoc5^WT^ mice (Fig. 2l, m). These results indicate that Exoc5 deletion in proximal tubule cells worsens renal fibrosis following UUO.

### Exoc5 knockout increases EMT following UUO

**Fig. 3:**
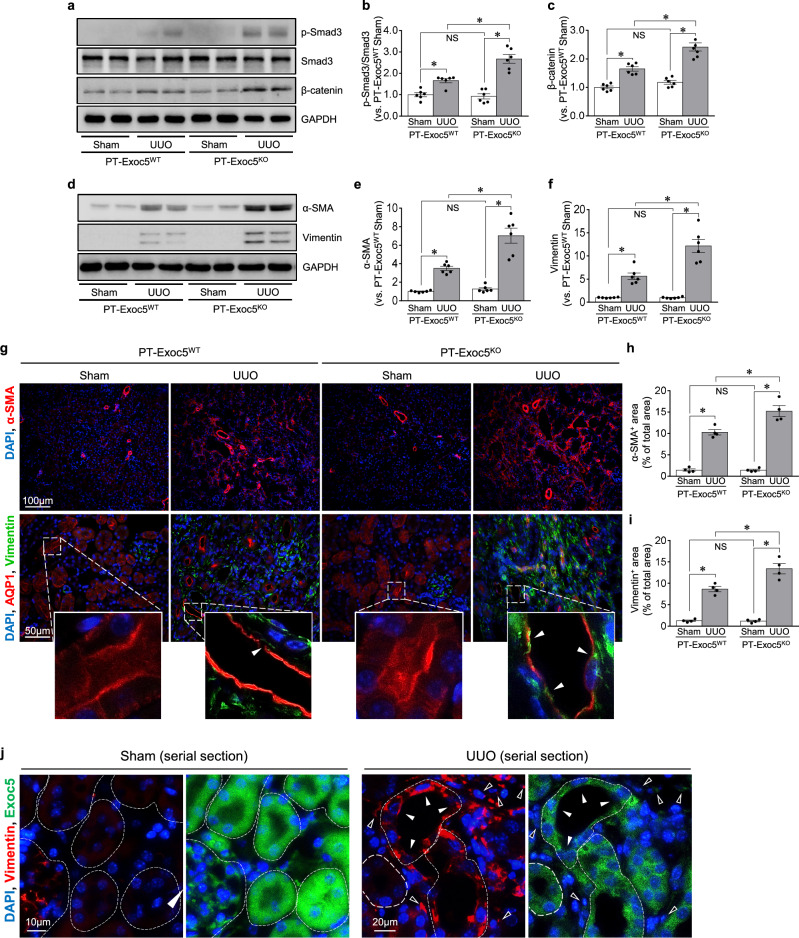

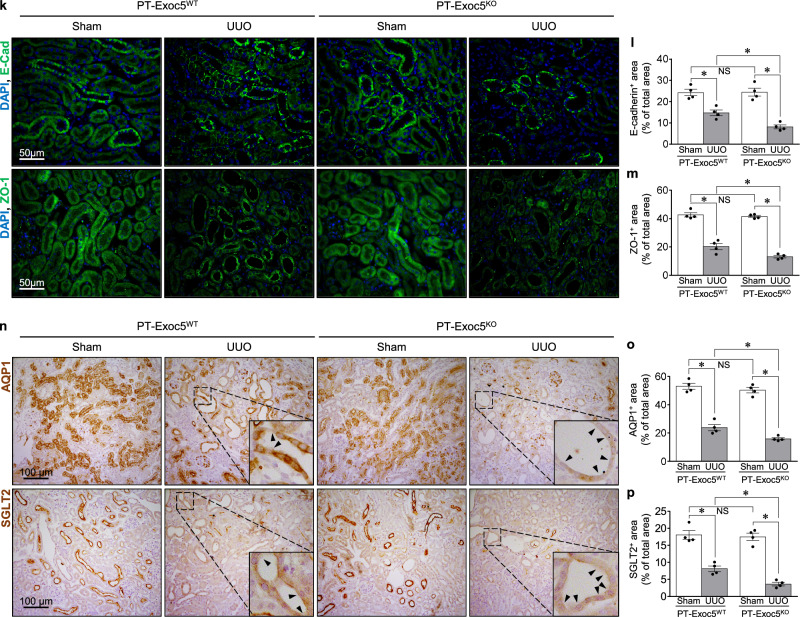
EMT progression in PT–Exoc5^WT^ and PT–Exoc5^KO^ mice following UUO. **a**, **d** Western blot of p-Smad3, Smad3 and β-catenin (**a**) and α-SMA and vimentin (**d**). **b**, **c**, **e**, **f** Graphs of p-Smad3/Smad3 (**b**), β-catenin (**c**), α-SMA (**e**) and vimentin (**f**) expression in the kidney tissue. GAPDH was used as the loading control, and densities of bands were quantified using ImageJ software. **g**, **j**, **k**, **n** Kidney sections were subjected to immunostaining using antibodies against α-SMA (red), vimentin (green in **g** and red in **j**), and Exoc5 (green), AQP1 (red in **g** and brown in **n**), E-cadherin (green), ZO-1 (green), and SGLT2 (brown). DAPI staining (blue) was used to visualize cell nuclei. The white arrowheads indicate vimentin positivity in tubular cells, and the black arrowheads indicate cells with apical loss of AQP1 or SGLT2 expression. In **j**, adjacent serial kidney sections are immunostained for Exoc5 (green) and vimentin (red). The filled arrowheads indicate vimentin-expressing tubular cells. The open arrowheads indicate interstitial cells co-expressing Exoc5 and vimentin. **h**, **i**, **l**, **m**, **o**, **p** The quantification was performed by measuring the area of α-SMA (**h**), vimentin (**i**), E-cadherin (**l**), ZO-1 (**m**), AQP1 (**o**) and SGLT2 positivity (**p**). PT–Exoc5^WT^ and PT–Exoc5^KO^ mice were subjected to either UUO or sham surgery, and kidneys were collected 7 days following surgery. Results are expressed as the mean ± s.e.m. (*n* = 4–6). *P < 0.05. NS, not significant.

As Exoc5 regulates epithelial cell polarity formation and differentiation^12,19,21,22,27^, we investigated whether Exoc5 affects UUO-induced EMT. UUO increased the phosphorylation of Smad3 (p-Smad3/Smad3) and the expression of β-catenin, transcription factors that contribute to the progression of fibrosis and EMT^28,29^ in the kidneys of both mice, with greater increases in PT–Exoc5^KO^ versus PT–Exoc5^WT^ mice (Fig. 3a–c). UUO also increased the expression of α-SMA, a component of the stress fibers of myofibroblasts and vimentin, a type III intermediate filament protein in mesenchymal cells, in both groups of mice, with greater increases seen in PT–Exoc5^KO^ compared with PT–Exoc5^WT^ mice (Fig. 3d–f). Consistent with western blot data, UUO increased the α-SMA-positive and vimentin-positive area in the interstitium with greater increases seen in PT–Exoc5^KO^ compared with PT–Exoc5^WT^ mice (Fig. 3g–i). When Exoc5 and vimentin expression were determined in wild-type mice, Exoc5 expression was reduced in vimentin-expressing tubular cells, and vimentin-positive interstitial cells co-expressed Exoc5 after UUO (Fig. 3j). These results indicate that fibroblasts express Exoc5 during fibrosis and that UUO decreases Exoc5 expression in tubules, whereas it increases vimentin at those sites, resulting in the loss of epithelial cell characteristics by UUO. In addition, UUO decreased the expression of E-cadherin and ZO-1, which are canonical markers of epithelial polarity and junctional proteins, in both groups, with greater decreases of them in PT–Exoc5^KO^ compared with PT–Exoc5^WT^ mice (Fig. 3k–m). We further investigated whether UUO induces epithelial polarity loss, that is, the loss of epithelial cell characteristics, by evaluating the expression pattern of proximal tubule transporters, AQP1 and SGLT2. AQP1 and SGLT2 are specifically expressed in proximal tubule cell apical membrane and are widely used as makers of proximal tubule cells^30,31^. In addition, several studies have shown that UUO induces the loss of apical expression of AQP1 and SGLT2 in proximal tubules^30,31^. Therefore, we evaluated the proximal tubule polarity by assessing AQP1 and SGLT2 expression. In the present study, we found that AQP1 and SGLT2 are prominently expressed in the apical membrane of the proximal tubule cells of sham-operated kidneys and that UUO induced the apical loss of AQP1 and SGLT2, with a greater loss in PT–Exoc5^KO^ compared with PT–Exoc5^WT^ mice (Fig. 3n–p). These results indicate that Exoc5 knockout exacerbates the UUO-induced apical loss of AQP1 and SGLT2, as well as the UUO-induced decrease in E-cadherin and ZO-1 expression.

### Exoc5 knockout increases Pax2 re-expression following UUO

**Fig. 4:**
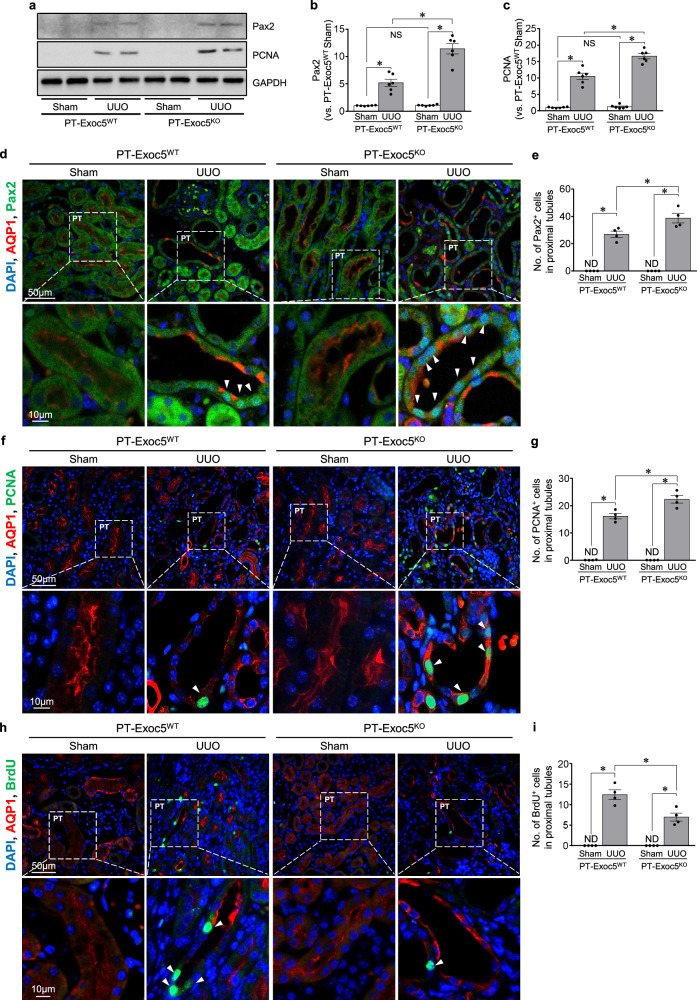

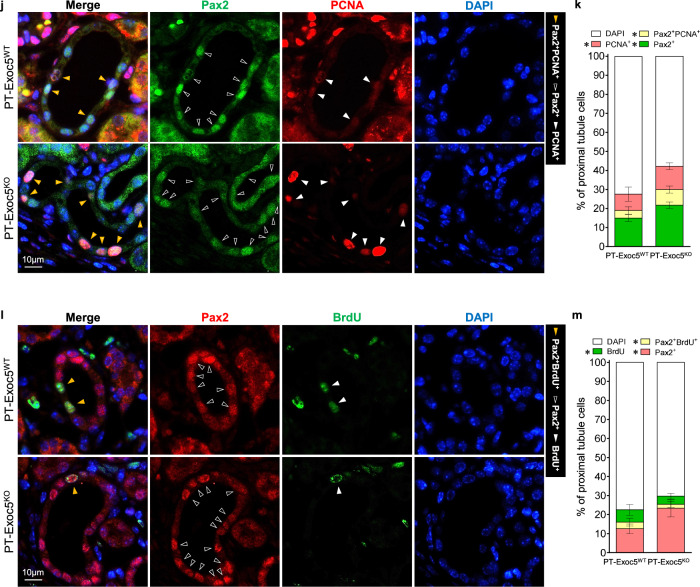
Pax2, PCNA and BrdU expression in the kidneys of PT–Exoc5^WT^ and PT–Exoc5^KO^ mice following UUO. **a** Western blot of Pax2 and PCNA expression. **b**, **c**, Graphs of Pax2 (**b**) and PCNA (**c**) expression in the kidney tissue; the densities of bands were quantified using ImageJ software (*n* = 6). GAPDH was used as the loading control. **d**, **f**, **h** The kidney sections were subjected to immunofluorescence staining using antibodies against Pax2 (green) and AQP1 (red) (**d**), PCNA (green) and AQP1 (red) (**f**), BrdU (green) and AQP1 (red) (**h**). DAPI staining (blue) was performed to visualize cell nuclei. Arrowheads indicate Pax2-, PCNA- and BrdU-positive nuclei in proximal tubule cells, respectively. **e**, **g**, **i** The quantification was performed by counting Pax2- (**e**), PCNA- (**g**) and BrdU-positive cells (**i**) in proximal tubules in a 40× micrograph with a field area of 0.1 mm^2^. **j**, **l** The kidney sections were subjected to immunofluorescence staining using antibodies against Pax2 (green) and PCNA (red) (**j**) and Pax2 (red) and BrdU (green) (**l**). DAPI staining (blue) was used to visualize cell nuclei. **k**, **m** The quantification was performed by counting Pax2- and PCNA-positive cells (**k**) and Pax2- and BrdU-positive cells (**m**) in proximal tubules in a 40× micrograph with a field area of 0.1 mm^2^. Pax2-, PCNA- and BrdU-positive cells in proximal tubules are marked by arrowheads, as indicated by the colors in the figures. PT–Exoc5^WT^ and PT–Exoc5^KO^ mice were subjected to either UUO or sham surgery, and kidneys were collected 7 days following the surgery. BrdU was administered every other day from the day before UUO until death. Results are expressed as the mean ± s.e.m. (*n* = 4–6). *P < 0.05. NS, not significant; ND, not detected.

Next, we investigated whether Exoc5 affects paired box 2 (Pax2, a member of the paired box family and nuclear transcription factor) re-expression. Pax2 plays a critical role in renal tubule cell differentiation and proliferation during kidney development and disappears from the proximal tubule cells after nephron maturation. Pax2 can be re-expressed during certain pathological conditions such as fibrosis and EMT^32,33^. Here, UUO induced Pax2 expression in both groups of mice, with greater expression in PT–Exoc5^KO^ compared with PT–Exoc5^WT^ mice (Fig. 4a, b). Pax2 was not detected in the proximal tubule cells of sham-operated mice (Fig. 4d, e), whereas Pax2 was detected in the nuclei of proximal tubular cells of UUO-operated kidneys of both groups of mice with a greater increase in PT–Exoc5^KO^ compared with PT–Exoc5^WT^ mice (Fig. 4d, e). Pax2 was mainly expressed in proximal tubule cells that had lost the apical localization of AQP1. AQP1 was expressed on the apical membrane of the proximal tubules on sham-operated mouse kidneys, whereas in UUO kidneys, the proximal tubule cells lost the typical apical pattern of AQP1 localization (Fig. 4d). These results indicate that UUO reactivates Pax2 expression and that Exoc5 knockout augments the reactivation, with concomitant cell dedifferentiation.

To further study cell differentiation and proliferation, we investigated PCNA (which is expressed during cell proliferation and in damaged cells after injury) and BrdU incorporation, which indicates dividing cells. UUO increased the PCNA expression and BrdU-positive cells in both groups of mice; however, the increase in PCNA-positive cells was greater in PT–Exoc5^KO^ than in PT–Exoc5^WT^ mice, whereas the increase in BrdU-positive cells was less in PT–Exoc5^KO^ than in PT–Exoc5^WT^ mice (Fig. 4a, c, f–i). UUO-induced Pax2 expression appeared in a majority of PCNA-positive cells, but only a minority of BrdU-positive cells appeared in both groups of mice (Fig. 4j–m). The population of PCNA-positive cells in Pax2-positive cells was higher in PT–Exoc5^KO^ compared with PT–Exoc5^WT^ mice (Fig. 4j, k), whereas the population of BrdU-positive cells in Pax2-positive cells was lower in PT–Exoc5^KO^ compared with PT–Exoc5^WT^ mice (Fig. 4l, m). Unlike PCNA, which is expressed in the G1-S phase as well as in damaged cells, BrdU is only incorporated in the S phase of mitosis^34,35^; therefore, the increased PCNA expression due to Exoc5 knockout probably reflects both increased cell damage and cell proliferation. These data suggest that Exoc5 knockout suppresses the cell cycle entry, that is, cell division, of dedifferentiated cells.

### Exoc5 knockout increases the expression and activation of YAP following UUO

**Fig. 5:**
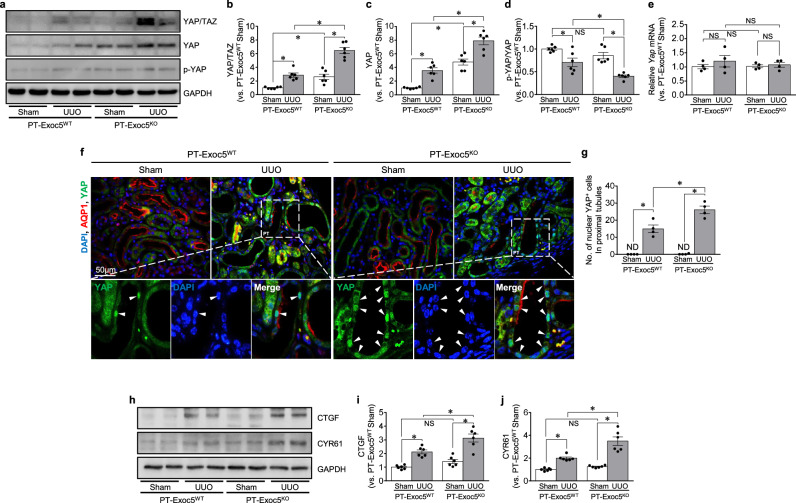
YAP expression in the kidneys of PT–Exoc5^WT^ and PT–Exoc5^KO^ mice following UUO. **a** Western blot of YAP/TAZ, YAP and p-YAP protein expression. **b**–**d** Graphs of YAP/TAZ (**b**) and YAP (**c**) protein expression in kidney tissue, and band densities were quantified using ImageJ software (*n* = 6). The p-YAP/YAP ratio was calculated on the basis of band intensity (**d**). **e** The *Yap* mRNA levels were measured by quantitative rtPCR (*n* = 4). **f** The kidney sections were subjected to immunofluorescence staining using antibodies against YAP (green) and AQP1 (red) antibodies. DAPI staining (blue) was performed to visualize cell nuclei. Arrowheads indicate YAP-positive nuclei in proximal tubule cells. **g** The quantification was performed by counting YAP-positive cells in proximal tubules in a 40× micrograph with a field area of 0.1 mm^2^. **h** Western blot of CTGF and CYR61 protein expression. **i**, **j** Graphs of CTGF (**i**) and CYR61 (**j**) protein expression in kidney tissue; band densities were quantified using ImageJ software (*n* = 6). GAPDH was used as the loading control. PT–Exoc5^WT^ and PT–Exoc5^KO^ mice were subjected to either UUO or sham surgery, and kidneys were collected 7 days following the surgery. Results are expressed as the mean ± s.e.m. (*n* = 4–6). *P < 0.05. NS, not significant.

As the exocyst complex is associated with the Hippo signaling pathway and YAP (a representative downstream component of the Hippo signaling pathway)^27,36,37^, which affects EMT^38,39^, we investigated whether Exoc5 is associated with YAP signaling pathway. Furthermore, YAP is also known to regulate Pax2 expression^40,41^. Interestingly, Exoc5 knockout alone increased the expression of YAP/TAZ, YAP and p-YAP compared with PT–Exoc5^WT^ mice following a sham operation (Fig. 5a–c). UUO increased the expression of YAP/TAZ and YAP, and the expression levels of these were greater in PT–Exoc5^KO^ compared with PT–Exoc5^WT^ mice (Fig. 5a–c). UUO decreased the ratio of p-YAP to YAP (p-YAP/YAP), which reflects YAP activation, in both groups and was lower in PT–Exoc5^KO^ compared with PT–Exoc5^WT^ mice (Fig. 5a, d). *Yap* messenger RNA (mRNA) levels were not significantly different between PT–Exoc5^WT^ and PT–Exoc5^KO^ mice in both sham and UUO groups (Fig. 5e). After UUO, but not the sham operation, YAP expression was observed in the nuclei of AQP1-positive proximal tubule cells in both groups along with decreased cytoplasmic YAP expression (Fig. 5f, g). This nuclear YAP-positive cell number was greater in PT–Exoc5^KO^ compared with PT–Exoc5^WT^ mice (Fig. 5f, g). In addition, UUO increased the expression of connective tissue growth factor (CTGF) and CYR61, products of YAP^42^, with greater increases in PT–Exoc5^KO^ compared with PT–Exoc5^WT^ mice (Fig. 5h–j). These data indicate that Exoc5 knockout alone upregulates YAP expression without a significant change in its mRNA expression and that Exoc5 knockout further augments UUO-induced YAP activation.

### Exoc5 KD increases YAP and accelerates EMT in HK-2 cells following TGF-β treatment

**Fig. 6:**
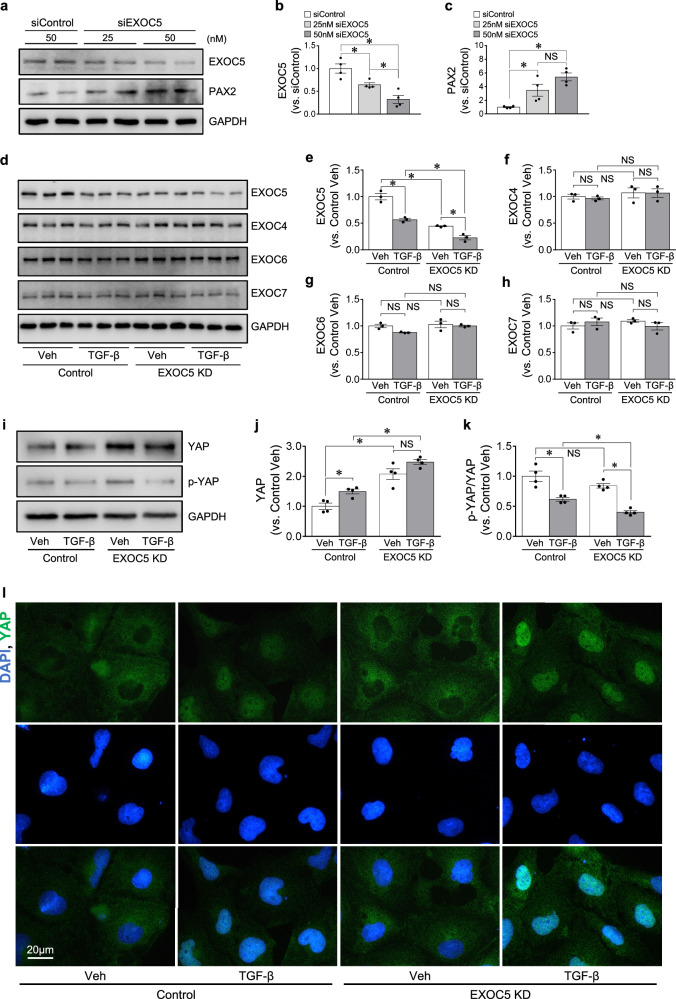

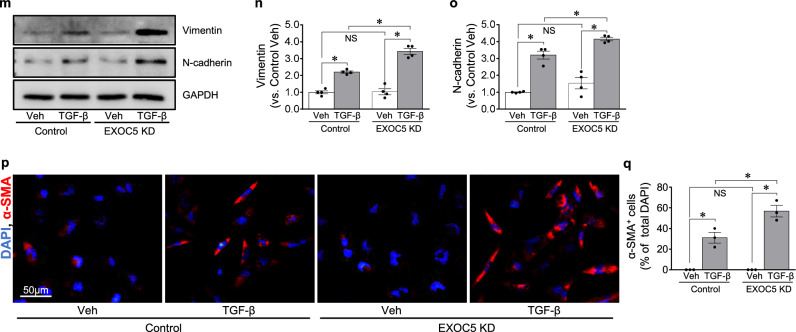
YAP expression and EMT progression in Exoc5-siRNA-treated HK-2 cells after TGF-β treatment. **a**–**k** Western blot (**a**, **d** and **i**) of EXOC5 (**b** and **e**), PAX2 (**c**), EXOC4 (**f**), EXOC6 (**g**), EXOC7 (**h**), YAP (**j**) expression in the cell lysate; band densities were quantified using ImageJ software (*n* = 3–4); The p-YAP/YAP ratio was calculated on the basis of band intensity (**k**). **l** The fixed cells were subjected to immunofluorescence staining using anti-YAP antibody (green). **m**–**o** Western blot (**m**) of the vimentin (**n**) and N-cadherin (**o**) expression in the cell lysate, analyzed 48 h after TGF-β treatment, and band densities were quantified using ImageJ software (*n* = 4). GAPDH was used as a loading control. **p**, **q** The fixed cells were subjected to immunofluorescence staining using anti-α-SMA antibody (red) (**p**), and α-SMA-positive cells were counted (**q**) in a 20× micrograph. DAPI staining (blue) was used to visualize cell nuclei. HK-2 cells were transfected with either Exoc5-siRNA or scrambled siRNA and the cells were treated with or without 5 ng/ml TGF-β for 1 or 48 h. Results are expressed as the mean ± s.e.m. (*n* = 3). *P < 0.05. NS, not significant.

Finally, we examined the effect of Exoc5 downregulation, using EXOC5 siRNA, on YAP expression and TGF-β-induced EMT in HK-2 cells, a human proximal tubule cell line. EXOC5 siRNA treatment significantly downregulated EXOC5 expression (Fig. 6a, b). In addition, the EXOC5 knockdown (EXOC5 KD) significantly increased PAX2 expression compared with control cells (Fig. 6a, c). The TGF-β treatment decreased EXOC5 expression in both EXOC5 KD and control cell groups (Fig. 6d, e). EXOC5 KD did not alter EXOC4, EXOC6 and EXOC7 expression, nor did TGF-β treatment alter the expression of these components (Fig. 6d–h). These results indicate that EXOC5 KD does not alter the expression of EXOC4, EXOC6 and EXOC7, suggesting that EXOC5 may affect TGF-β-induced EMT without altering the expression of other exocyst components, at least of EXOC4, EXOC6 and EXOC7. The EXOC5 KD alone significantly increased YAP expression and TGF-β-induced reduction of p-YAP/YAP ratio was greater in EXOC5 KD compared with control (Fig. 6i–k). The immunofluorescence staining also showed that TGF-β treatment induced nuclear YAP expression in both groups, with higher nuclear expression in EXOC5 KD compared with control cells (Fig. 6l). These results indicate that the reduction of EXOC5 expression increases YAP and PAX2 expression, and it further exacerbates TGF-β-induced YAP activation.

Next, we investigated whether EXOC5 KD affects TGF-β-induced EMT. EXOC5 KD did not significantly change the expression of vimentin and N-cadherin (Fig. 6m–o). The TGF-β treatment increased the expression of vimentin and N-cadherin in both groups, and these increases were greater in EXOC5 KD compared with control cells (Fig. 6m–o). In addition, the TGF-β treatment increased α-SMA-positive cells in both groups, with a greater increase in EXOC5 KD compared with control cells (Fig. 6p, q). These results indicate that the EXOC5 downregulation in HK-2 cells increases YAP expression and augments TGF-β-induced YAP activation and EMT.

## Discussion

Our findings demonstrate that proximal tubule cell-specific Exoc5 deficiency exacerbates kidney fibrosis accompanied by increased EMT and enhanced YAP activation following UUO. Consistently, Exoc5 downregulation in HK-2 cells exacerbates TGF-β-induced EMT. These findings indicate that Exoc5 plays an important role in the progression of kidney fibrosis, suggesting that targeting Exoc5 may be a novel therapeutic strategy for resolving kidney fibrosis.

The loss of Exoc5 disrupts organogenesis in various tissues, including the kidney^15–17,37^. The Mouse Genome Informatics (https://www.informatics.jax.org/) and GenitoUrinary Development Molecular Anatomy Project (https://gudmap.org/) databases show that Exoc5 mRNA and PEPCK (*Pck1*) mRNA are expressed in renal proximal tubule cells at the E15.5 stage. This indicates that Exoc5 knockout in proximal tubules by crossing *Exoc5*^*f/f*^ mice with *PEPCK-Cre* mice occurs during the development of proximal tubules. In the present study, we found that Exoc5 knockout in proximal tubules does not induce significant morphological and functional changes in the kidneys of mice as compared with PT–Exoc5^WT^ mice. However, Exoc5 knockout exacerbates UUO-induced fibrosis. Furthermore, Exoc5 downregulation in HK-2 cells by siRNA accelerates the TGF-β-induced loss of cellular polarity and YAP expression, resulting in increased features of EMT. These data indicate that Exoc5 deficiency in the renal proximal tubules does not cause defects in kidney structure, localization of transporters and kidney function, but Exoc5 is a pivotal factor in the progression of renal fibrosis. Unlike other renal cells, at least in the present study using *PEPCK-Cre* mice, Exoc5-knockout mice showed normal kidney development and function. Therefore, targeting Exoc5 to the proximal tubule may be a strategy for developing therapeutics for kidney diseases. Research on Exoc5 overexpression or direct delivery may provide novel therapeutic insights for the treatment of kidney disease such as fibrosis.

It has been demonstrated that the exocyst complex regulates a variety of cellular functions^9,27,43^. In the present study, we found that Exoc5 KD in cultured human renal proximal tubule cells exacerbates TGF-β-induced EMT, and Exoc5 knockout in mouse proximal tubule cells exacerbates renal fibrosis induced by UUO as reflected by a loss of polarity and an increase of vimentin expression. Furthermore, we found that in wild-type mice, UUO decreases Exoc5 expression, along with increased vimentin expression and proximal tubule cell polarity loss. These results suggest that Exoc5 plays an important role in preventing the transition of mature, damaged and/or repaired epithelial cells to mesenchymal cells. Other studies have demonstrated that individual components of the exocyst complex play an important role in EMT. Lu et al. reported that Exoc7 participates in the EMT process of cancer cells through isoform switching and the abnormal Exoc7 isoform activates the expression of Snail and ZEB2 which leads to a transition between epithelial and mesenchymal phenotypes^44^. By contrast, Qian et al. reported that Exoc1 knockdown in A549 cells, a human lung carcinoma epithelial cell, prevents TGF-β-induced EMT by the inhibition of Akt phosphorylation^45^. Tanaka et al. reported that the expression of Exoc4, but not Exoc3 or Exoc7, increases after TGF-β treatment and mediates EMT by increasing N-cadherin and Smad3/4 expression through CREB-binding protein regulation^46^. To further define the association of other exocyst components with fibrosis, we investigated the expression of Exoc4, an exocyst subcomplex 1 component, and Exoc6 and Exoc7, exocyst subcomplex 2 components, with fibrosis. In the present study, we found that Exoc5 knockout and Exoc5 KD, in the absence of UUO and TGF-β treatment, did not alter the expression of Exoc4, Exoc6 and Exoc7. However, both UUO in mice and TGF-β treatment in HK-2 cells altered these component expressions; UUO decreased the expression of Exoc4 and Exoc5 but not Exoc7, with no differences between PT–Exoc5^KO^ and PT–Exoc5^WT^ mice. In HK-2 cells, TGF-β treatment reduced Exoc5 but not Exoc4, Exoc6 and Exoc7. These results indicate that Exoc5 may affect UUO- or TGF-β-induced EMT with no significant alteration in the expression of other exocyst components, at least Exoc4, Exoc6 and Exoc7. The different changes in Exoc4 expression between UUO-subjected kidney and TGF-β-treated HK-2 cells may be attributed to the heterogeneous cell populations and the presence of Exoc5 on those populations in kidney lysates. In the present study, the reduction of Exoc5 may exacerbate kidney fibrosis, without the change of Exoc4, Exoc6 and Exoc7, suggesting that Exoc5 plays an important role in kidney fibrosis progression. However, to define the role of other exocyst complex components on kidney fibrosis, further studies modulating each exocyst component are required.

The inappropriate dedifferentiation of renal tubule epithelial cells to mesenchymal cells during the repair following injury is a major cause of renal fibrosis^47^. Pax2, an essential transcription factor for cell differentiation, is expressed throughout the kidney, including the proximal tubule, during kidney development^48^. Pax2 regulates the conversion of metanephric mesenchyme precursor cells to fully differentiated renal tubule epithelial cells in the early stages of embryonic development^49,50^, and then, Pax2 is downregulated as cells leave the mitotic cycle, that is, when the renal tubule cells are fully differentiated^51^. It is known that the abnormal expression of Pax2 during kidney development impedes normal nephrogenesis^49^. Recent studies have shown that Pax2 is re-expressed in mature kidneys under certain pathological conditions, including renal fibrosis^50^. Injured and repairing phases, which occur during organ fibrosis, increase Pax2 expression, and Pax2 overexpression induces EMT in renal tubule epithelial cells^32,33,52^. Furthermore, Dressler and Woolf reported that the misexpression of Pax2 is associated with the initiation of progression of renal disease^51^. Here, we found that UUO induces Pax2 expression along with a loss of polarity of AQP1 in renal tubule cells and that Pax2 expression in PT–Exoc5^KO^ mice is greater than that in PT–Exoc5^WT^ mice. We also found that the percentage of PCNA^+^ cells among Pax2^+^ cells was higher in PT–Exoc5^KO^ mice, suggesting that the Pax2 re-expression in stressed cells is greater in PT–Exoc5^KO^ mice. In addition, Fig. 4m shows that the percentage of BrdU^+^ cells among Pax2^+^ cells was lower in PT–Exoc5^KO^ mice, indicating that Exoc5 knockout suppresses the proliferation of Pax2^+^ dedifferentiated cells. Supporting this, in human proximal tubule cells, the downregulation of Exoc5 increased Pax2 expression. Therefore, we speculate that Exoc5 knockout augments UUO-induced Pax2 induction, thereby activating renal tubule epithelial cell dedifferentiation to mesenchymal cells rather than cell proliferation. To define how Exoc5 regulates Pax2 expression in renal tubule cells, further studies are required.

It has been well established that YAP activation causes EMT and fibrosis, whereas YAP inhibition prevents fibrosis, indicating that YAP is involved in kidney diseases^38,39,53,54^. YAP activation induces organ fibrosis by promoting EMT^53^. Jin et al. reported that the pharmacological inhibition of YAP using verteporfin ameliorates UUO-induced renal tubular injury and fibroblast activation, thereby inhibiting renal fibrosis^54^. In addition, recent studies have demonstrated the association between YAP and the exocyst complex. Zaman et al. reported that during viral infection Exoc8 activates Hippo signaling by inhibiting YAP^36^. Lobo et al. reported that Exoc5 knockout in zebrafish upregulates phosphorylated MOB1 (p-MOB1), one of the proteins that regulates the Hippo signaling pathway^37^. In the present study, we found that Exoc5 deletion in proximal tubule cells alone increased YAP expression without a significant change in its mRNA expression and that Exoc5 deletion promotes more YAP activation after UUO through the YAP/p-YAP ratio and translocation to the nucleus. In addition, the Exoc5 deletion enhanced the expression of CTGF, which is a direct target gene of YAP and activates EMT^55–57^. CTGF is known to contribute to the progression of fibrosis through the proliferation of fibroblasts and EMT activation in various cell types such as renal tubule epithelial cells^56,57^. These results suggest that Exoc5 inhibits YAP activation, resulting in decreased CTGF production and reduced fibrosis.

Our previous studies demonstrated that Exoc5 interacts genetically and biochemically with the small GTPase Cdc42^58,59^. YAP and Cdc42 are mutually regulated, and Cdc42 deletion increases YAP expression^41,60,61^. YAP is also a negative regulator of primary cilia, formation of which are regulated by the exocyst^62,63^. Therefore, increased YAP expression following Exoc5 deletion may be a complex biological consequence of the interaction between the exocyst and YAP. However, it is also possible that Exoc5 degrades cytoplasmic YAP. In addition, it has been reported that *Pax* family genes utilize YAP and TAZ as coactivators to produce Pax proteins. In fact, YAP or TAZ expression increases Pax family protein expression by more than tenfold^64^. Tanigawa et al. reported that YAP downregulation decreases Pax2 and concomitantly increases E-cadherin, causing a mesenchymal-to-epithelial transition^40^. Reginensi et al. reported that YAP deletion in renal mesenchymal progenitor cells downregulates Pax2 expression and inhibits nephron formation^41^. Therefore, we speculate that Exoc5 deletion activates YAP, thereby leading to Pax2 re-expression and the acceleration of EMT and fibrosis.

Although our study clearly shows that Exoc5 deficiency exacerbates kidney fibrosis accompanied by proximal tubule EMT and YAP activation, the current study has several limitations that should be addressed in future studies. First, the present study did not examine the effects of Exoc5 deficiency on fibroblasts; the functional role of Exoc5 in fibroblasts remains incompletely defined. Considering that fibroblasts are a major contributor to the progression of kidney fibrosis, further studies using fibroblast-specific Exoc5 deletion mice are necessary to clarify cell type-specific mechanisms and to more precisely define the therapeutic potential of Exoc5 modulation. Second, although the association between Exoc5 and YAP is supported by our data, definitive mechanistic evidence remains lacking because YAP rescue studies were not performed in the current study. Therefore, rescue experiments involving the pharmacologic or genetic modulation of YAP remain important research objectives. Third, to assess the therapeutic potential of Exoc5 in fibrosis, approaches involving Exoc5 overexpression, gene and protein delivery or pharmacological modulation should be investigated. Future studies addressing these limitations could strengthen the findings of the current study and provide direct evidence for the association between Exoc5–YAP and kidney fibrosis.

## Supplementary information

Supplementary Information

## Data Availability

All datasets generated for this study are included in the Article/Supplementary Information. Supplementary Information accompanies the manuscript on the *Experimental and Molecular Medicine* website (http://www.nature.com/emm/).

## References

[1] Rockey, D. C., Bell, P. D. & Hill, J. A. Fibrosis-a common pathway to organ injury and failure. *N. Engl. J. Med.* **373**, 1138–1149 (2015). 10.1056/NEJMra1300575 25785971

[2] Jun, J. I. & Lau, L. F. Resolution of organ fibrosis. *J. Clin. Invest.* **128**, 97–107 (2018). 29293097 10.1172/JCI93563 PMC5749507

[3] Thiery, J. P., Acloque, H., Huang, R. Y. & Nieto, M. A. Epithelial–mesenchymal transitions in development and disease. *Cell* **139**, 871–890 (2009). 19945376 10.1016/j.cell.2009.11.007

[4] Carew, R. M., Wang, B. & Kantharidis, P. The role of EMT in renal fibrosis. *Cell Tissue Res.* **347**, 103–116 (2012). 21845400 10.1007/s00441-011-1227-1

[5] Liu, Y. Renal fibrosis: new insights into the pathogenesis and therapeutics. *Kidney Int.* **69**, 213–217 (2006). 16408108 10.1038/sj.ki.5000054

[6] el Nahas, A. M., Muchaneta-Kubara, E. C., Essawy, M. & Soylemezoglu, O. Renal fibrosis: insights into pathogenesis and treatment. *Int. J. Biochem. Cell Biol.* **29**, 55–62 (1997). 9076941 10.1016/s1357-2725(96)00119-7

[7] Novick, P., Field, C. & Schekman, R. Identification of 23 complementation groups required for post-translational events in the yeast secretory pathway. *Cell* **21**, 205–215 (1980). 6996832 10.1016/0092-8674(80)90128-2

[8] TerBush, D. R., Maurice, T., Roth, D. & Novick, P. The exocyst is a multiprotein complex required for exocytosis in Saccharomyces cerevisiae. *EMBO J.* **15**, 6483–6494 (1996). 8978675 PMC452473

[9] Mei, K. & Guo, W. The exocyst complex. *Curr. Biol.* **28**, R922–R925 (2018). 30205058 10.1016/j.cub.2018.06.042

[10] Pereira, C. et al. The exocyst complex is an essential component of the mammalian constitutive secretory pathway. *J. Cell Biol.* **222**, e202205137 (2023). 10.1083/jcb.202205137 PMC10041652 36920342

[11] Grindstaff, K. K. et al. Sec6/8 complex is recruited to cell–cell contacts and specifies transport vesicle delivery to the basal–lateral membrane in epithelial cells. *Cell* **93**, 731–740 (1998). 9630218 10.1016/s0092-8674(00)81435-x

[12] Zuo, X., Guo, W. & Lipschutz, J. H. The exocyst protein Sec10 is necessary for primary ciliogenesis and cystogenesis in vitro. *Mol. Biol. Cell* **20**, 2522–2529 (2009). 19297529 10.1091/mbc.E08-07-0772 PMC2682593

[13] Zuo, X. et al. Exo70 interacts with the Arp2/3 complex and regulates cell migration. *Nat. Cell Biol.* **8**, 1383–1388 (2006). 17086175 10.1038/ncb1505

[14] Bryant, D. M. et al. A molecular network for de novo generation of the apical surface and lumen. *Nat. Cell Biol.* **12**, 1035–1045 (2010). 20890297 10.1038/ncb2106 PMC2975675

[15] Fulmer, D. et al. Defects in the exocyst–cilia machinery cause bicuspid aortic valve disease and aortic stenosis. *Circulation* **140**, 1331–1341 (2019). 31387361 10.1161/CIRCULATIONAHA.119.038376 PMC6989054

[16] Fogelgren, B. et al. Urothelial defects from targeted inactivation of exocyst Sec10 in mice cause ureteropelvic junction obstructions. *PLoS ONE* **10**, e0129346 (2015). 26046524 10.1371/journal.pone.0129346 PMC4457632

[17] Nihalani, D. et al. Disruption of the exocyst induces podocyte loss and dysfunction. J. Biol. Chem. 294, 10104–10119 (2019). 10.1074/jbc.RA119.008362 PMC6664173 31073028

[18] Fogelgren, B. et al. Exocyst Sec10 protects renal tubule cells from injury by EGFR/MAPK activation and effects on endocytosis. *Am. J. Physiol. Renal Physiol.* **307**, F1334–F1341 (2014). 25298525 10.1152/ajprenal.00032.2014 PMC4269694

[19] Park, K. M. et al. Exocyst Sec10 protects epithelial barrier integrity and enhances recovery following oxidative stress, by activation of the MAPK pathway. *Am. J. Physiol. Renal Physiol.* **298**, F818–F826 (2010). 20053792 10.1152/ajprenal.00596.2009 PMC2838587

[20] Zuo, X. et al. Cilia-deficient renal tubule cells are primed for injury with mitochondrial defects and aberrant tryptophan metabolism. Am. J. Physiol. Renal Physiol. 327, F61–F76 (2024). 10.1152/ajprenal.00225.2023 PMC11390130 38721661

[21] Lipschutz, J. H. et al. Exocyst is involved in cystogenesis and tubulogenesis and acts by modulating synthesis and delivery of basolateral plasma membrane and secretory proteins. *Mol. Biol. Cell* **11**, 4259–4275 (2000). 11102522 10.1091/mbc.11.12.4259 PMC15071

[22] Lipschutz, J. H. The role of the exocyst in renal ciliogenesis, cystogenesis, tubulogenesis, and development. *Kidney Res. Clin. Pract.* **38**, 260–266 (2019). 31284362 10.23876/j.krcp.19.050 PMC6727897

[23] Jang, H. S., Noh, M. R., Ha, L., Kim, J. & Padanilam, B. J. Proximal tubule cyclophilin D mediates kidney fibrogenesis in obstructive nephropathy. *Am. J. Physiol. Renal Physiol.* **321**, F431–F442 (2021). 34396791 10.1152/ajprenal.00171.2021 PMC8560409

[24] Park, K. M., Kim, J. I., Ahn, Y., Bonventre, A. J. & Bonventre, J. V. Testosterone is responsible for enhanced susceptibility of males to ischemic renal injury. *J. Biol. Chem.* **279**, 52282–52292 (2004). 15358759 10.1074/jbc.M407629200

[25] Park, K. M., Kramers, C., Vayssier-Taussat, M., Chen, A. & Bonventre, J. V. Prevention of kidney ischemia/reperfusion-induced functional injury, MAPK and MAPK kinase activation, and inflammation by remote transient ureteral obstruction. *J. Biol. Chem.* **277**, 2040–2049 (2002). 11696540 10.1074/jbc.M107525200

[26] Han, S. J. et al. Mitochondrial NADP^+^-dependent isocitrate dehydrogenase deficiency exacerbates mitochondrial and cell damage after kidney ischemia–reperfusion injury. *J. Am. Soc. Nephrol.* **28**, 1200–1215 (2017). 27821630 10.1681/ASN.2016030349 PMC5373447

[27] Zuo, X. et al. The exocyst acting through the primary cilium is necessary for renal ciliogenesis, cystogenesis, and tubulogenesis. *J. Biol. Chem.* **294**, 6710–6718 (2019). 30824539 10.1074/jbc.RA118.006527 PMC6497951

[28] Yamazaki, K. et al. Upregulated SMAD3 promotes epithelial-mesenchymal transition and predicts poor prognosis in pancreatic ductal adenocarcinoma. *Lab. Invest.* **94**, 683–691 (2014). 24709776 10.1038/labinvest.2014.53

[29] Sanchez-Tillo, E. et al. beta-catenin/TCF4 complex induces the epithelial-to-mesenchymal transition (EMT)-activator ZEB1 to regulate tumor invasiveness. *Proc. Natl Acad. Sci. USA* **108**, 19204–19209 (2011). 22080605 10.1073/pnas.1108977108 PMC3228467

[30] Li, C., Wang, W., Knepper, M. A., Nielsen, S. & Frokiaer, J. Downregulation of renal aquaporins in response to unilateral ureteral obstruction. *Am. J. Physiol. Renal Physiol.* **284**, F1066–F1079 (2003). 12517734 10.1152/ajprenal.00090.2002

[31] Zhou, W. et al. The gut microbe *Bacteroides fragilis* ameliorates renal fibrosis in mice. *Nat. Commun.* **13**, 6081 (2022). 36241632 10.1038/s41467-022-33824-6 PMC9568537

[32] Imgrund, M. et al. Re-expression of the developmental gene Pax-2 during experimental acute tubular necrosis in mice 1. *Kidney Int.* **56**, 1423–1431 (1999). 10504494 10.1046/j.1523-1755.1999.00663.x

[33] Maeshima, A., Maeshima, K., Nojima, Y. & Kojima, I. Involvement of Pax-2 in the action of activin A on tubular cell regeneration. *J. Am. Soc. Nephrol.* **13**, 2850–2859 (2002). 12444203 10.1097/01.asn.0000035086.93977.e9

[34] Gratzner, H. G. Monoclonal antibody to 5-bromo- and 5-iododeoxyuridine: a new reagent for detection of DNA replication. *Science* **218**, 474–475 (1982). 7123245 10.1126/science.7123245

[35] Essers, J. et al. Nuclear dynamics of PCNA in DNA replication and repair. *Mol. Cell. Biol.* **25**, 9350–9359 (2005). 16227586 10.1128/MCB.25.21.9350-9359.2005 PMC1265825

[36] Zaman, A. et al. Exocyst protein subnetworks integrate Hippo and mTOR signaling to promote virus detection and cancer. *Cell Rep.* **36**, 109491 (2021). 34348154 10.1016/j.celrep.2021.109491 PMC8383154

[37] Lobo, G. P. et al. The exocyst is required for photoreceptor ciliogenesis and retinal development. *J. Biol. Chem.* **292**, 14814–14826 (2017). 28729419 10.1074/jbc.M117.795674 PMC5592663

[38] Szeto, S. G. et al. YAP/TAZ are mechanoregulators of TGF–β–Smad signaling and renal fibrogenesis. *J. Am. Soc. Nephrol.* **27**, 3117–3128 (2016). 26961347 10.1681/ASN.2015050499 PMC5042658

[39] Liang, M. et al. Yap/Taz deletion in Gli^+^ cell-derived myofibroblasts attenuates fibrosis. *J. Am. Soc. Nephrol.* **28**, 3278–3290 (2017). 28768710 10.1681/ASN.2015121354 PMC5661271

[40] Tanigawa, S., Sharma, N., Hall, M. D., Nishinakamura, R. & Perantoni, A. O. Preferential propagation of competent SIX2^+^ nephronic progenitors by LIF/ROCKi treatment of the metanephric mesenchyme. *Stem Cell Rep.* **5**, 435–447 (2015). 10.1016/j.stemcr.2015.07.015 PMC4618653 26321142

[41] Reginensi, A. et al. Yap- and Cdc42-dependent nephrogenesis and morphogenesis during mouse kidney development. *PLoS Genet.* **9**, e1003380 (2013). 23555292 10.1371/journal.pgen.1003380 PMC3605093

[42] Wang, K. C. et al. Flow-dependent YAP/TAZ activities regulate endothelial phenotypes and atherosclerosis. *Proc. Natl Acad. Sci. USA* **113**, 11525–11530 (2016). 27671657 10.1073/pnas.1613121113 PMC5068257

[43] Tanaka, T., Goto, K. & Iino, M. Diverse functions and signal transduction of the exocyst complex in tumor cells. *J. Cell. Physiol.* **232**, 939–957 (2017). 27669116 10.1002/jcp.25619

[44] Lu, H. et al. Exo70 isoform switching upon epithelial–mesenchymal transition mediates cancer cell invasion. *Dev. Cell* **27**, 560–573 (2013). 24331928 10.1016/j.devcel.2013.10.020 PMC3908839

[45] Qian, X. et al. Sec3 knockdown inhibits TGF-β induced epithelial-mesenchymal transition through the down-regulation of Akt phosphorylation in A549 cells. *Biochem. Biophys. Res. Commun.* **519**, 253–260 (2019). 31495494 10.1016/j.bbrc.2019.08.145

[46] Tanaka, T., Goto, K. & Iino, M. Sec8 modulates TGF-β induced EMT by controlling N-cadherin via regulation of Smad3/4. *Cell Signal.* **29**, 115–126 (2017). 27769780 10.1016/j.cellsig.2016.10.007

[47] Ferenbach, D. A. & Bonventre, J. V. Mechanisms of maladaptive repair after AKI leading to accelerated kidney ageing and CKD. *Nat. Rev. Nephrol.* **11**, 264–276 (2015). 25643664 10.1038/nrneph.2015.3 PMC4412815

[48] Dressler, G. R., Deutsch, U., Chowdhury, K., Nornes, H. O. & Gruss, P. Pax2, a new murine paired-box-containing gene and its expression in the developing excretory system. *Development* **109**, 787–795 (1990). 1977574 10.1242/dev.109.4.787

[49] Torres, M., Gomez-Pardo, E., Dressler, G. R. & Gruss, P. Pax-2 controls multiple steps of urogenital development. *Development* **121**, 4057–4065 (1995). 8575306 10.1242/dev.121.12.4057

[50] Song, J., Chen, X., Zhang, L., Song, D. & Xiong, H. MicroRNA-204-3p modulates epithelial–mesenchymal transition by targeting paired box gene 2 in human melanoma A-375 cells. *Transl. Cancer Res.* **8**, 2032–2043 (2019). 35116952 10.21037/tcr.2019.09.10 PMC8798729

[51] Dressler, G. R. & Woolf, A. S. Pax2 in development and renal disease. *Int. J. Dev. Biol.* **43**, 463–468 (1999). 10535325

[52] Li, L., Wu, Y. & Yang, Y. Paired box 2 induces epithelial-mesenchymal transition in normal renal tubular epithelial cells of rats. *Mol. Med. Rep.* **7**, 1549–1554 (2013). 23503776 10.3892/mmr.2013.1365

[53] Noguchi, S., Saito, A. & Nagase, T. YAP/TAZ signaling as a molecular link between fibrosis and cancer. *Int. J. Mol. Sci.* **19,** 3674 (2018). 10.3390/ijms19113674 PMC6274979 30463366

[54] Jin, J. et al. Inhibition of Yes-associated protein by verteporfin ameliorates unilateral ureteral obstruction-induced renal tubulointerstitial inflammation and fibrosis. *Int. J. Mol. Sci.* **21,** 8184 (2020). 10.3390/ijms21218184 PMC7662854 33142952

[55] Pan, D. The Hippo signaling pathway in development and cancer. *Dev. Cell* **19**, 491–505 (2010). 20951342 10.1016/j.devcel.2010.09.011 PMC3124840

[56] Sonnylal, S. et al. Connective tissue growth factor causes EMT-like cell fate changes in vivo and in vitro. *J. Cell Sci.* **126**, 2164–2175 (2013). 23525012 10.1242/jcs.111302 PMC3672936

[57] Lipson, K. E., Wong, C., Teng, Y. & Spong, S. CTGF is a central mediator of tissue remodeling and fibrosis and its inhibition can reverse the process of fibrosis. *Fibrogen. Tissue Repair* **5**, S24 (2012). 10.1186/1755-1536-5-S1-S24 PMC3368796 23259531

[58] Choi, S. Y. et al. Cdc42 deficiency causes ciliary abnormalities and cystic kidneys. *J. Am. Soc. Nephrol.* **24**, 1435–1450 (2013). 23766535 10.1681/ASN.2012121236 PMC3752951

[59] Zuo, X., Fogelgren, B. & Lipschutz, J. H. The small GTPase Cdc42 is necessary for primary ciliogenesis in renal tubular epithelial cells. *J. Biol. Chem.* **286**, 22469–22477 (2011). 21543338 10.1074/jbc.M111.238469 PMC3121392

[60] Sakabe, M. et al. YAP/TAZ-CDC42 signaling regulates vascular tip cell migration. *Proc. Natl Acad. Sci. USA* **114**, 10918–10923 (2017). 28973878 10.1073/pnas.1704030114 PMC5642684

[61] Zhang, Z. et al. CDC42 controlled apical–basal polarity regulates intestinal stem cell to transit amplifying cell fate transition via YAP–EGF–mTOR signaling. *Cell Rep.* **38**, 110009 (2022). 35021092 10.1016/j.celrep.2021.110009 PMC8826493

[62] Rausch, V. & Hansen, C. G. The Hippo pathway, YAP/TAZ, and the plasma membrane. *Trends Cell Biol.* **30**, 32–48 (2020). 31806419 10.1016/j.tcb.2019.10.005

[63] Kim, J. et al. Actin remodelling factors control ciliogenesis by regulating YAP/TAZ activity and vesicle trafficking. *Nat. Commun.* **6**, 6781 (2015). 25849865 10.1038/ncomms7781

[64] Manderfield, L. J. et al. Pax3 and Hippo signaling coordinate melanocyte gene expression in neural crest. *Cell Rep.* **9**, 1885–1895 (2014). 25466249 10.1016/j.celrep.2014.10.061 PMC4267159

