# Supplementary material for: Deficiency of exocyst complex component Exoc5 exacerbates the progression of kidney fibrosis
Source: Exp Mol Med. 2026 Mar 4;58(3):681–95. doi: 10.1038/s12276-026-01649-8 (PMC13049091; doi:10.1038/s12276-026-01649-8)
Supplement: Supplementary file 1 — Supplementary Information [file 12276_2026_1649_MOESM1_ESM.pdf]

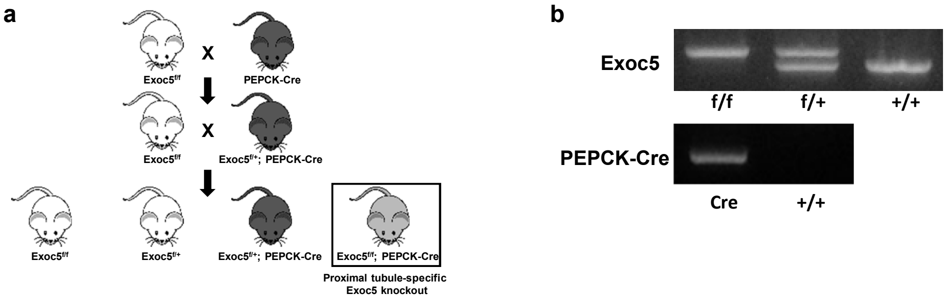

Supplementary Fig. 1. Generation of PT–Exoc5^{KO} mice. **a** Cartoon of the PT–Exoc5^{KO} mice breeding strategy. **b** Mice genotypes were determined by PCR from tail genomic DNA using the following primers: Exoc5 loxP (Forward: 5'-GCCTGTAACTCACAGAGATC-3'. Reverse: 5'-GCTGGCATTCTAAGTCATGG-3') and PEPCK Cre (Forward: 5'-CGGTGCTAACCAGCGTTTTC-3'. Reverse: 5'-TGGGCGGCATGGTGCAAGTT-3').
